# Fostering mental health and chronic diseases self-management among professional truck drivers

**DOI:** 10.1093/eurpub/ckac130.120

**Published:** 2022-10-25

**Authors:** D Desrosier, ME Carrière, S Chartrand, MV Gaudet, J Jbilou

**Affiliations:** 1 Psychology, Université de Moncton, Moncton, Canada; 2 School of Medicine, Université de Sherbrooke, Moncton, Canada

## Abstract

Most passengers and goods in Canada travel by road. The trucking industry is the backbone of the tangible goods economy. However, the health and well-being of this aging workforce is in jeopardy. Recent data reveals that 86% of the truckers’ community are 50 years old and over, 4% are female and 5% are immigrants. Moreover, 75% of the male truckers self-reported one or more health condition (54% obesity, 19% hyperlipidemia, 18 % high blood pressure, 11 % type II diabetes). Despite, the high prevalence of risk factors (e.g. stress, depression/anxiety, lower level of education, social isolation and financial challenges) and preventable chronic diseases among truckers, in New Brunswick and elsewhere in Canada, there is a lack of on-the-road accessible lifestyle change programs. Therefore, tailored interventions are needed to appropriately support them adopt healthy behaviors. Using the Re-AIM Framework, we carried out 23 semi-structured interviews to inform the development of tailored educational material. The aims were: to describe the needs and challenges and to design a truckers-sensitive educational intervention. The theoretical foundation of this qualitative study is articulated around concepts extracted from cognitive and behavioural theories (transtheoretical model of behaviour change). Qualitative analysis of verbatims identified four major themes: Lifestyle challenges, Social and individual representation of healthy behaviors, Health education strategies and communication and Motivational and engagement strategies. Drawing upon these findings we developed tailored educational material and pre-validated them with a small group of professional truck drivers. Our findings informed the development of an educational intervention to support truckers manage and improve their mental health and self-management of chronic diseases. The next step is to implement a randomized clinical trial to test and assess acceptability, feasibility, and effectiveness of our intervention.

**Key messages:**

- Improving mental health of professional truck drivers is an urgent public health issue.
- Format and content of mental health and chronic disease self-management need to be adapted at the cognitive (health lieracy) and capacity (resources, time) level.

